# Supplementary material for: Exploring the risks of over-reliance on AI in diagnostic pathology. What lessons can be learned to support the training of young pathologists?
Source: PLoS One. 2025 Aug 28;20(8):e0323270. doi: 10.1371/journal.pone.0323270 (PMC12393786; doi:10.1371/journal.pone.0323270)
Supplement: Attachment — Submitted filename: Response_to_Reviewers_auresp_2.docx [file pone.0323270.s006.docx]

**Rebuttal letter**

PONE-D-24-53739

**AI in diagnostic pathology: exploring the risks of over-reliance and its clinical consequences. What lessons can be learned to support the training of young pathologists?**

Pr Cécile BADOUAL, MD, PhD

Paris, 04/03/2025

Dear Editor, dear Reviewers,

We would like to thank you for your insightful comments that helped to improve our article.

We are pleased to present a new version in which we hope to address the concerns raised by Reviewer 1. The last revisions are in green font (previous revisions were kept in blue font).

**Reviewer #1 :**

**I still think the authors should focus still more on addressing how to avoid the errors. There is no proof provided whatsoever that shows the effect of training to mitigate the problem. I think this is a flaw. It should be addressed, at least in the text, and at least how this kind of training should be done.**

Thank you for your remark. In our study, we did not directly demonstrate that training younger and non-specialized pathologists on the panel would enhance their ability to use AI effectively and improve diagnostic accuracy. To establish this, a repeated evaluation of the slides by pathologists would be necessary to observe whether their performance improves through iterative learning from errors. However, our dataset is limited, particularly concerning misinterpreted cases. This raises the concern that pathologists may recall their previous incorrect diagnoses and provide correct answers through memory recall rather than genuine improvement in diagnostic skills, in the same way an AI model can be overfit on limited datasets. Such investigations would be more appropriately conducted in a longitudinal study, tracking pathologists from the initial stages of their training through their early years of board-certified practice.

In radiology, a field with substantial AI integration in daily practice, previous studies have indicated that younger radiologists are particularly prone to automation bias, resulting in an over-reliance on AI predictions (1), (2), (3). A study by Mehrizi et al. (4) suggested that even increased explainability does not mitigate this issue. Our results suggest the same problem applies to pathology. Specifically, in cases where AI led to incorrect diagnoses despite accurate unassisted reviews, the more experienced pathologists considered the AI's input but did not follow it uncritically. In contrast, recently board-certified and non-specialized pathologists were more likely to rely on the AI's suggestions, which may show that this tendency to automated bias in younger practitioners might be related to the “figure of authority” described in cognitive development science, here consisting in a mathematical algorithm, which operating mode can seem not understandable, and thus not really questionable, when lacking a strong opinion and confidence from the beginning (5). These considerations underscore the need for alternative approaches to assist pathologists in using AI effectively.

To address these challenges, radiology societies in North America have introduced structured educational programs, such as the National Imaging Informatics Course – Radiology and the Radiological Society of North America Imaging AI Certificate (6), (7). These courses teach how AI models work and their ability to induce biased judgments. Pathology societies are starting to follow a similar trajectory, offering AI-focused training that emphasizes the recognition of AI limitations and the risks associated with bias (8). In France, the integration of a dedicated AI mandatory course is quite recent (2023), and it has yet to include a specific course on AI biases.

In our opinion, the confidence score can be of great use to raise awareness on AI limitations on specific cases. Evaluating its influence on pathologist training outcomes should require a dedicated, long-term study.

These elements were integrated to the revised manuscript (text in green – lines 344-386).

Nota Bene : we noticed an error in Fig4 (wrong image for 4A), the new figure was added to the submitted files.

**References :**

1.	Tejani AS, Ng YS, Xi Y, Rayan JC. Understanding and Mitigating Bias in Imaging Artificial Intelligence. RadioGraphics. 2024 May 1;44(5):e230067.

2.	Bernstein MH, Atalay MK, Dibble EH, Maxwell AWP, Karam AR, Agarwal S, et al. Can incorrect artificial intelligence (AI) results impact radiologists, and if so, what can we do about it? A multi-reader pilot study of lung cancer detection with chest radiography. Eur Radiol. 2023 Jun 2;33(11):8263–9.

3.	Dratsch T, Chen X, Rezazade Mehrizi M, Kloeckner R, Mähringer-Kunz A, Püsken M, et al. Automation Bias in Mammography: The Impact of Artificial Intelligence BI-RADS Suggestions on Reader Performance. Radiology. 2023 May 1;307(4):e222176.

4.	Rezazade Mehrizi MH, Mol F, Peter M, Ranschaert E, Dos Santos DP, Shahidi R, et al. The impact of AI suggestions on radiologists’ decisions: a pilot study of explainability and attitudinal priming interventions in mammography examination. Sci Rep. 2023 Jun 7;13(1):9230.

5.	Mills CM. Knowing when to doubt: Developing a critical stance when learning from others. Dev Psychol. 2013 Mar;49(3):404–18.

6.	Tejani AS, Elhalawani H, Moy L, Kohli M, Kahn CE. Artificial Intelligence and Radiology Education. Radiol Artif Intell. 2023 Jan 1;5(1):e220084.

7.	Duong MT, Rauschecker AM, Rudie JD, Chen PH, Cook TS, Bryan RN, et al. Artificial intelligence for precision education in radiology. Br J Radiol. 2019 Nov;92(1103):20190389.

8.	Vos S, Hebeda K, Milota M, Sand M, Drogt J, Grünberg K, et al. Making Pathologists Ready for the New Artificial Intelligence Era: Changes in Required Competencies. Mod Pathol. 2025 Feb;38(2):100657.
